# Simulating Clinical Psychiatry for Medical Students: a Comprehensive Clinic Simulator with Virtual Patients and an Electronic Medical Record System

**DOI:** 10.1007/s40596-017-0860-8

**Published:** 2017-11-30

**Authors:** Yoshihito Matsumura, Hideto Shinno, Takahiro Mori, Yu Nakamura

**Affiliations:** Kagawa University School of Medicine, Miki, Kagawa Japan

**Keywords:** Psychiatry, Medical students, Simulator, Virtual patients, Computer-based learning

## Abstract

**Objective:**

A number of programs representing virtual patients for use in teaching settings have been developed in the field of psychiatry; however, they simulate only the interview process, not the entire scope of treatment. The authors have developed software through which students can experience the practice of psychiatry (in particular, with dementia patients) in its entirety. This study compares this software with conventional learning methods.

**Method:**

The control group was 43 fifth-year medical students in 2014 who studied using a conventional learning method (taking lectures and being in contact with actual patients). The experimental group was 36 fifth-year medical students in 2015 that used computer software (taking lectures and with reduced time in contact with actual patients). The authors compared the two groups. Each group was tested before and after clinical training on their acquisition of knowledge of dementia. The control group was tested in 2014, and the experimental group was tested in 2015.

**Results:**

The difference in average test scores between the two groups was statistically significant (*p* = 0.01), with the experimental group scoring higher.

**Conclusions:**

The results indicate that students who were taught using a computer-based software method were better able to answer a standard series of questions designed to evaluate their understanding of dementia than those who were taught in a conventional manner.

This study demonstrated that there is a possibility to improve education in the field of psychiatry using a comprehensive clinic simulator.

The use of simulated teaching materials, such as virtual reality simulators and mannequin-based simulation, is increasing in a variety of medical fields, including surgery, pediatrics, obstetrics, and anesthesia [1–4], because of the limitations of conventional methods of instruction in these fields. In medical training, the treatment of patients must receive the highest priority, and the education of medical students takes place in the remaining time available. In addition, the instructing physician must ensure that the patient does not suffer because of the student; for this reason, the physician must place restrictions on students’ activities. Moreover, students are not always able to experience all types of cases that would be suitable for their learning within the clinical training period. Finally, some patients may indicate that they do not wish to be interviewed by a student, making it difficult for the student to learn from the specifics of that particular case.

To solve these problems, the learning process has shifted toward the use of standardized patients (SPs) [5], or people who act as if they have certain diseases. While invaluable to the teaching process, the use of SPs is limited by the cost of employing these individuals and their ability to accurately exhibit the intricacies of the disease that they are attempting to demonstrate. Further, students and SPs must coordinate their learning times [6], which can be another obstacle.

Simulation-based teaching materials are a third option. This approach eliminates the inconvenience of using SPs while making it possible for students to effectively acquire clinical abilities [7] without placing any actual patients at a disadvantage. Such materials can provide an opportunity for students to learn safely about sensitive, important, or rare cases [8]. Computer simulation and virtual patients (VPs) are currently used for the acquisition of skills such as history-taking, clinical decision-making, leadership, and teamwork [9]. Although VPs have been developed for certain psychiatric disorders, including major depression, bipolar disorder [10], post-traumatic stress disorder [11], and conduct disorder [12], the selection of VPs is not very diverse and there are limited teaching materials aimed at psychiatry students.

Learners have only been able to interview VPs for a few years. Previously, VPs were not capable of asking questions to the learners about their disease, the tests they must undergo, or the drugs prescribed to them. Hence, the questioning between the learners and the VPs was unidirectional. There are now some existing VPs [13] that can answer questions from learners and ask questions back. Almost all real patients will question the physician about the disease from which they are suffering and the medical care that they will receive. In real medical practice, physicians must give proper, respectful explanations to patients on a daily basis. We considered it necessary to create simulated teaching materials that were closer to real medical practice and that can recreate such scenarios.

Until now, there have only rarely been simulated teaching materials that students can use to experience medical practice in its entirety. Therefore, we developed a comprehensive clinic simulator. The word “entirety” here means all activities done in the examination room.

The primary purpose of this study is to learn whether the developed software is more effective than the educational method prevalent thus far in the acquisition of knowledge of dementia within the field of psychiatry.

The secondary purpose of this study is to learn how much the motivation for learning changes before and after using the software.

## Methods

### Subjects

In all, 36 fifth-year medical students in psychiatric clinical training at the Kagawa University in Japan between April and July 2015 were selected as our experimental subjects. As our control group, we used 43 fifth-year medical students under clinical training in the previous year (between April and July 2014) who had acquired their knowledge of psychiatry through conventional means. To reduce the likelihood that the results would be affected by the different periods of study, the months when the two groups of students did their clinical training were matched. In Japan, students enter medical school after graduating from high school. “Fifth-year medical student” means that it is the fifth year after the high school graduation. Students are 22 years old or older.

CBT (computer-based testing) and OSCE (Objective Structured Clinical Examination) are conducted on a common platform across the country before clinical training, and clinical training is not permitted unless the score is above a certain level. The average CBT grade of the control group was 62.68 (IRT). IRT is Item Response Theory. IRT is useful for considering the difficulty of multiple tests as equal (e.g., comparing the results of tests conducted at different times). The average CBT grade of the experiment group was 62.62 (IRT). There was no significant difference in the CBT score (IRT) between the control group and the experiment group (*p* = 0.98 > 0.05). The average OSCE marks of the control group and the experiment group were 86.79 and 86.80, respectively. There was no significant difference in the OSCE score between the control group and the experiment group (*p* = 0.99 > 0.05). The average age of the control group and the experiment group was 23.9 and 24.1 years respectively. There was no significant difference in the age of the students (*p* = 0.84 > 0.05).

There was no major change in the curriculum of other medical subjects between the two groups. In the curriculum of this medical school, neither the control group nor the experiment group had ever used software such as the simulator developed by us.

Before beginning the study, we informed the students how we intended to use the data from our tests and questionnaire. We also explained that they would not be disadvantaged in any way if they declined to participate. All subjects gave written consent to their participation.

### Method Overview

We compared our clinic simulator with conventional learning methods in knowledge acquisition pre- and post-intervention.

The control group had contact with actual patients (63 h) and attended lectures (11 h) during the rotation period. The experimental group used a simulator (0.75 h) and had contact with actual patients (62.25 h) as well as VPs and attended lectures (11 h) during the rotation period. The time to contact with the actual patient was reduced and the remaining time was used for the VP experience. The aim of the VP program is ensure that students get an experience of each of four typical dementia types. Both groups were lectured on psychiatry (11 h), including dementia (2 h), with the same content and time. Although the time that control students are in the ward is guaranteed, there is no assurance that they will encounter four types of dementia patients. Depending only on the experience gained in a real ward, one student can come into contact with four types of dementia several times, while another does not come across the ailment at all. So the experience in the real ward has been partially (0.75 h) altered to the virtual experience.

In addition, the motivation of the experimental group (36 medical students) who used the software was compared before and after their experience. Since motivation is not constantly measured, it was evaluated before and after using the software.

## Materials

### Development

We developed this simulation software independently (rather than in collaboration with a software company) to ensure a high degree of flexibility, reduce costs, and overcome the limitations of existing products. When a for-profit company produces software, considerable time and capital are required to make even small improvements.

We considered the following to be essential features: the learner (functioning in the role of the physician) can interview patients and their families, read their facial expressions during the interview, run tests, give diagnoses, and give prescriptions; patients and their families can ask questions about the disease from which the patient is suffering, the tests that the patient may undergo, and the prescriptions that the patient may receive; learners can respond to these questions; and orders for tests and prescriptions can be conducted using a realistic electronic medical record system.

We created the software using the Action Script of Adobe Flash Professional CS6 Version 12.0.0.481. Anyone with the ability to work with this program would be able to create the same software.

### Software Environment

This simulator can run in an internet web browser. However, for the purposes of this study, subjects used a stand-alone system, because we wanted to examine how students used the software before making it available on the internet.

The software ran on the Windows 7 operating system (Microsoft Corporation). The internet browser used to run the software was Internet Explorer 9.010 (Microsoft Corporation), and the version of Adobe Flash Player used was 18.0.0.194.

### Starting the Software

After we launched the software, students had the option of watching a short video that explains how to use it. The comprehensive clinic simulator is constructed to look like the electronic medical record system used by physicians at the Kagawa University Hospital. Students log in to the simulator, entering an ID and password in a process that is similar to the one followed by physicians at the hospital. As in a real electronic medical records system, only the VP’s name, age, and gender are initially displayed. No other information is available regarding the VPs, just as when actual physicians first meet their patients. Clinical practice begins from this point.

### Patient Types Included in the Software Training Suite

**Fig. 1:**
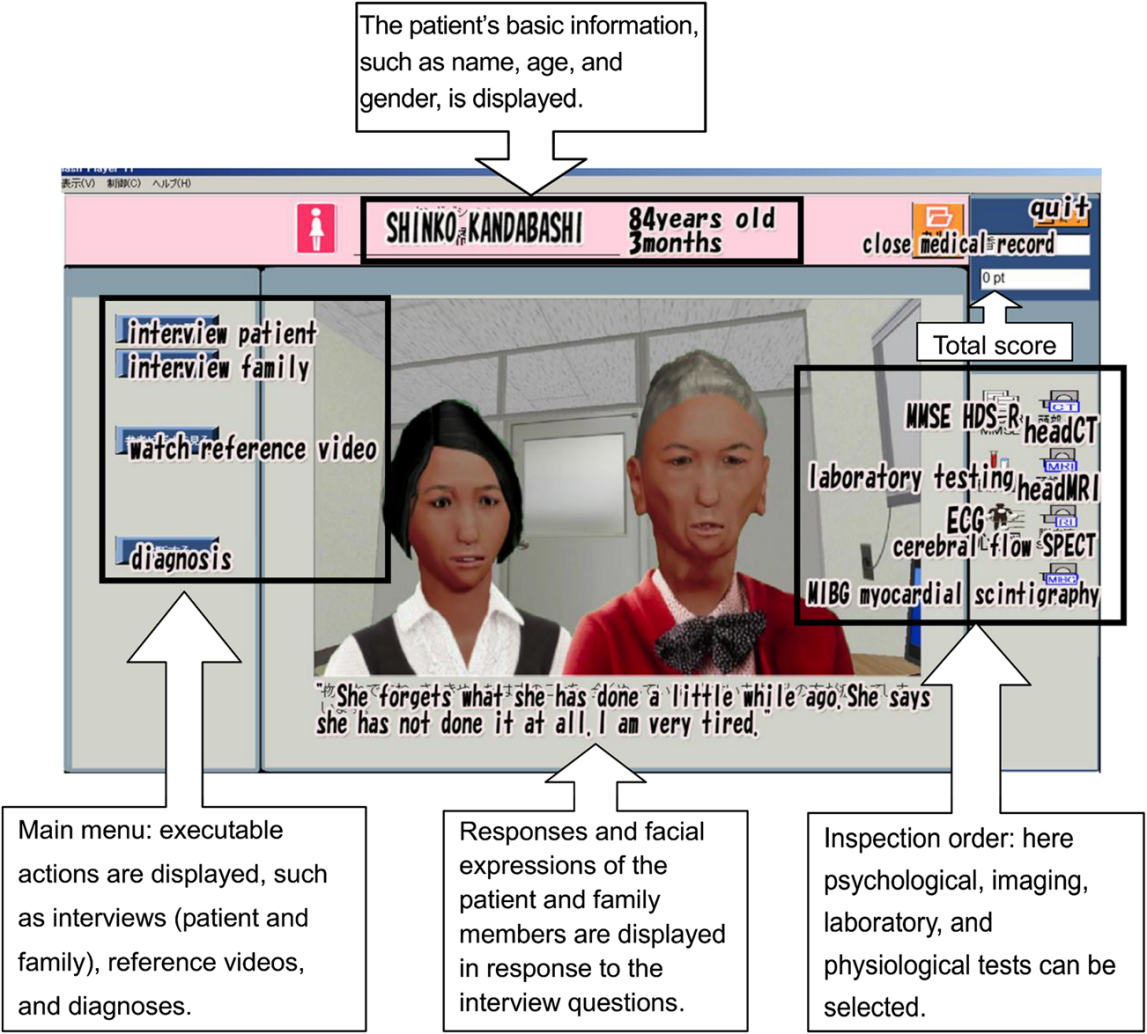
Screenshot of the comprehensive clinic simulator. The ordering system is similar to an electronic medical record system. Test results, prescriptions, and other functions, such as the virtual doctor, are displayed in response to the learner’s selections

We initially created four VPs with dementia for the study. They included an 84-year-old woman with dementia of Alzheimer type, a 73-year-old woman with dementia with Lewy bodies, a 77-year-old man with frontotemporal lobar degeneration, and a 73-year-old man with vascular dementia. When the student clicked on a line with a VP’s name, selecting it from the patient list on the computer screen, the VP and his or her family details appeared rendered in a simulated 3D examination room (Fig. 1).

### Examination Procedure

The names of four VPs appeared on the screen. Students clicked one VP’s name. Students were required to interview VPs with the virtual family members present, but they had no information on the VP or the family members before beginning the examination, just as a real doctor would.

Students choose what they want to hear from the listed options. Neurological examinations are included in these interview options. If students press a button, the examination is done automatically. Image inspections, blood tests, and the like are made possible by pushing the button of the examination ordered in the same way as the menu of the electronic medical record system.

**Fig. 2:**
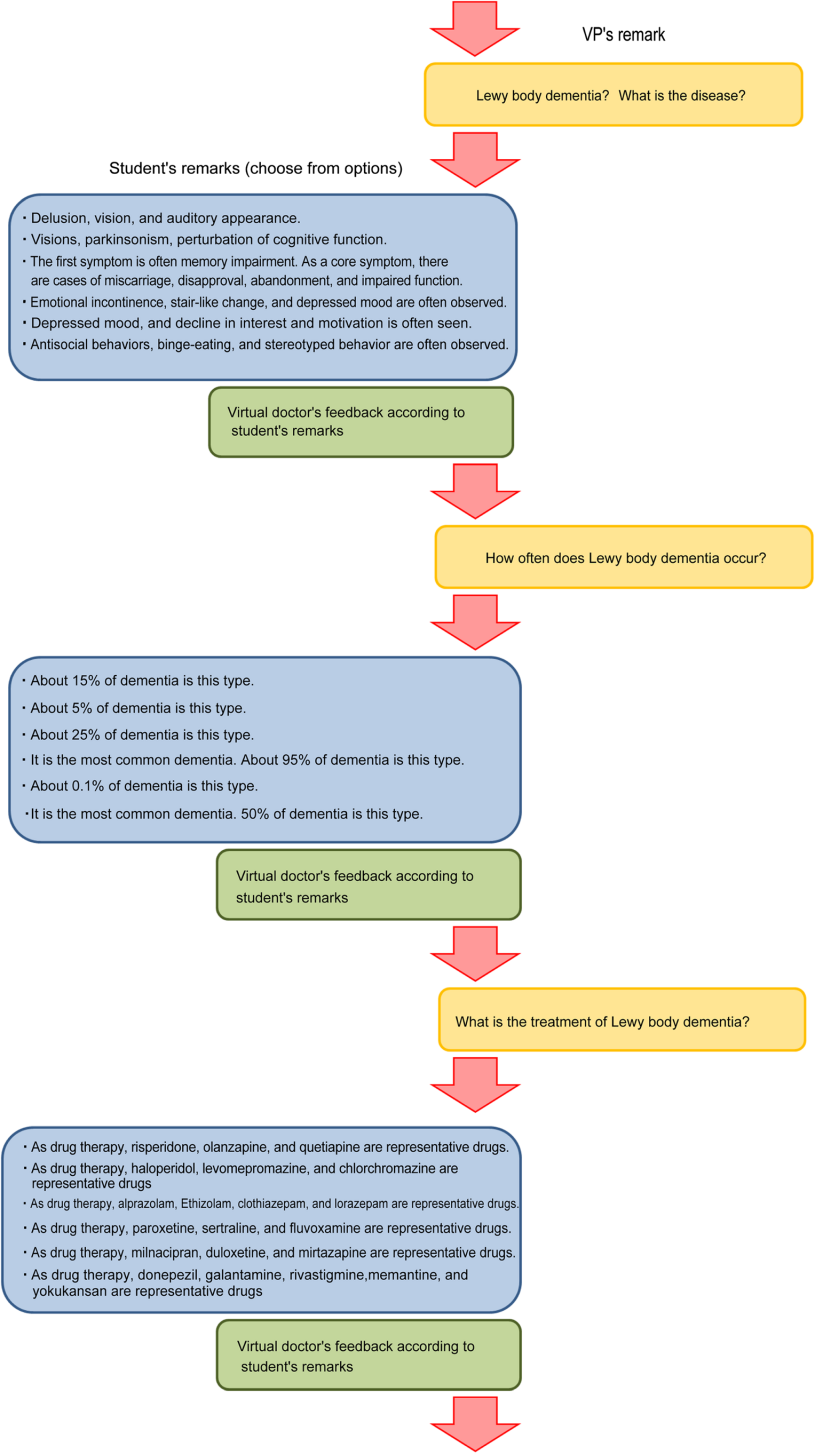
The interaction between patients and students. When a student selects a diagnosis, the VP asks what kind of symptoms the disease presents. After the student selects the answer to this question, the VP asks whether the disease is common

VPs and their families changed their facial expressions. They asked the student (playing the role of the physician) questions related to the VP’s illness, tests, and prescriptions. In some cases, they asked further follow-up questions after the student answered the initial question (Fig. 2).

The menu that the student-physician used to issue orders to test the patient was also devised to resemble the electronic medical records system at the Kagawa University Hospital. All of the test images (head CT, head MRI, cerebral blood flow SPECT) and electrocardiograms were original. They resembled those of actual patients, but no data of actual patients were used here or elsewhere in the simulator.

### Feedback to Students

**Fig. 3:**
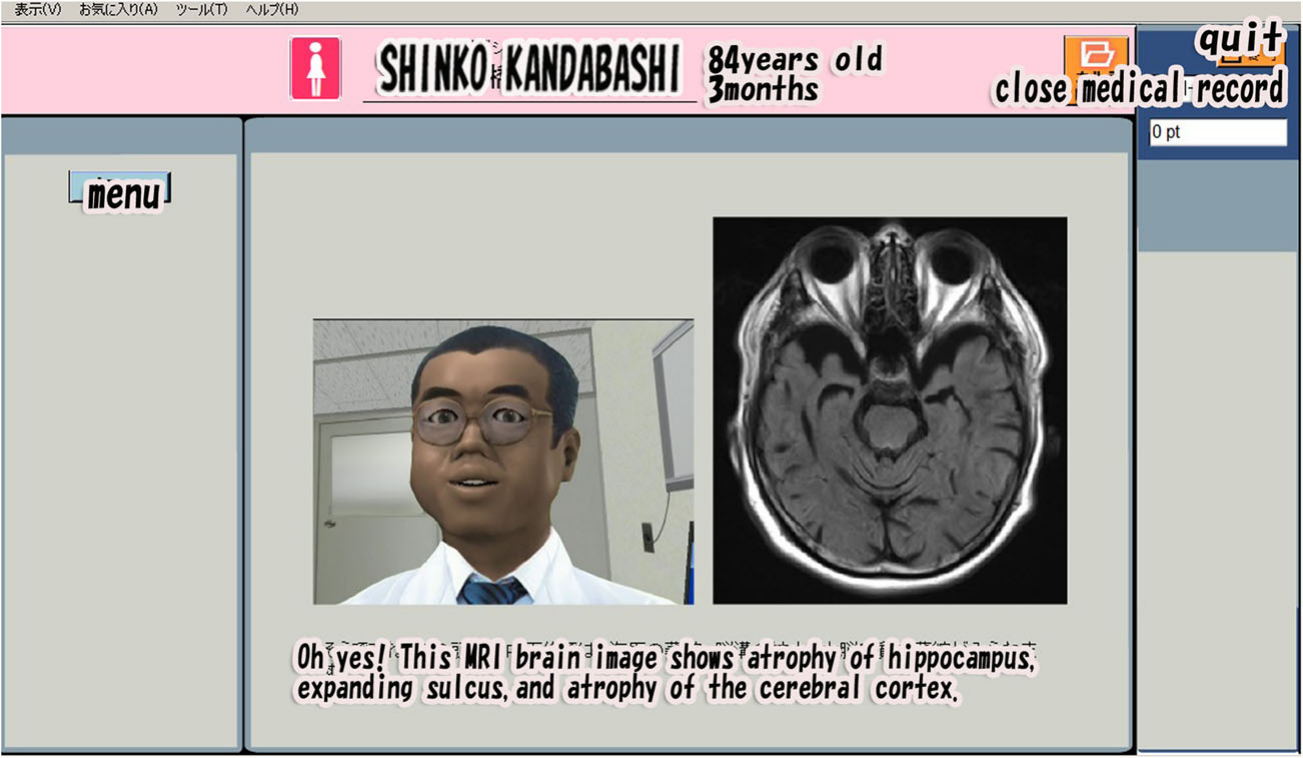
Screenshot showing a virtual doctor giving feedback. When the student has completed some form of action (examination, diagnosis, explanation, prescription, etc.), a virtual doctor appears to give feedback

When the students completed some form of action (examination, diagnosis, explanation, prescription, etc.), a virtual doctor appeared and gave advice. The virtual doctor provided a commentary, which included an indication of whether the student’s behavior was correct (Fig. 3). All input into the system is by the student’s pressing of a button. The virtual doctor can thus judge whether the act of the student is correct by the button that is pressed. Almost all feedback was given by the software. Students used the software with a teacher nearby. When the computer could not address an issue, humans compensated directly.

## Outcome Measures

### Pre-intervention and Post-intervention Tests

To evaluate the students’ acquisition of psychiatric knowledge, we created tests that require inputting keywords. Students answered questions by entering 27 keywords. One point was awarded for each correct input. The range of possible scores is 0–27. Neither the VP simulator nor the knowledge test count for a grade. There is no word group list at the time of the test. Instead of selecting keywords from a word group, students wrote keywords that they themselves conceived. The test examined basic knowledge of dementia. The contents of the test include “core and peripheral symptoms of dementia,” “image inspection and psychological examination necessary for the diagnosis of dementia,” “drugs for dementia,” “symptoms of dementia of Alzheimer type,” “symptoms of Lewy body dementia,” “symptoms of cerebrovascular dementia,” and “symptoms of frontotemporal lobar dementia.” This is all included in the content that students study in clinical psychiatry rotation. Traditionally, students hope to learn these items from actual patients. However, if the actual patient refuses contact with the student or if actual patients with the disease do not appear in the student’s rotation period, the students have to learn the content without a relevant patient experience.

The content validity of the tests was confirmed by two physicians who specialize in the field of psychiatry. When the test was conducted with medical students, Cronbach’s *α*, which measures the internal consistency of tests, was 0.72 (*n* = 158), as each of the 79 students in the two groups took the test twice). A value of Cronbach’s *α* above 0.7 is satisfactory for a two-group comparison [14].

### Questionnaire Based on the ARCS Motivation Model

To evaluate the motivation of the medical students, we used a questionnaire based on the Attention, Relevance, Confidence, and Satisfaction (ARCS) motivation model, which is used when designing, developing, evaluating, or improving teaching materials. One study has stated that the “ARCS Model offers an approach for diagnosing students’ motivational issues while using computer-based instruction” [15]. The ARCS model includes four main categories: attention, relevance, confidence, and satisfaction [16]. In the Japanese-language version of the questionnaire associated with the ARCS motivation model, each of the four main ARCS categories has four sub-categories. Thus, the questionnaire consists of 16 sub-categories, each of which asks for ratings on a 9-point scale. The degree of motivation for each of the 16 categories is indicated through the selection of a rating (from 1 to 9) between two words or phrases that have opposite meanings (for example, “boring” and “interesting” or “likely to be used immediately” and “not likely to be used immediately”) [17]. The sub-categories of attention are “boring” and “interesting,” “felt sleepy” and “did not feel sleepy,” “vague curiosity” and “curiosity aroused,” and “not stimulating” and “variable and stimulating.” The sub-categories of relevance are “not worthwhile” and “worthwhile,” “no relevance to me” and “relevant to me,” “do not want to acquire” and “want to acquire,” and “learning process was not fun” and “learning process was fun.” The sub-categories of confidence are “I was not confident” and “I gained confidence,” “objective was vague” and “objective was clear,” “steady progress was impossible” and “steady progress was possible,” and “not creative in learning” and “was creative in learning.” The sub-categories of satisfaction are “dissatisfied” and “satisfied,” “not readily applicable” and “readily applicable,” “effort was not recognized” and “effort was recognized,” and “evaluation not consistent” and “evaluation was consistent.” The word “motivation” is used here to mean a process that initiates action and maintains and adjusts toward a target function.

We measured how the software affects the motivation for learning in general. If, after being used, the motivation for learning is lower than before the software is used, it means that it is not suitable as teaching material. Before measuring motivation, students take 10 h of lectures during the practice period. Therefore, the students can answer the question “I gained confidence” as recorded above.

### System Usability Scale

This study examined whether the simulator was effective in helping students to acquire psychiatric knowledge (of dementia) in comparison with traditional learning methods and in motivating them. However, we also measured the system’s usability to enable the future improvement of the simulator. We used the System Usability Scale (SUS) for this purpose. The SUS, which is widely used to measure comprehensive usability, requests answers to a series of statements on a 5- or 7-point Likert scale ranging from “strongly agree” to “strongly disagree.” The questions are divided into 10 categories, and the total scores in each category are converted into a range between 0 and 100 points [18, 19]. A score below 50 points indicates that the system is unacceptable; a score of at least 70 points represents acceptability. Marginal scores (i.e., between 50 and 70) are divided into “low marginal” and “high marginal” [20]. We translated the SUS into Japanese for use with the participants of this study. The Japanese version of SUS that we used had a 5-point Likert scale. The scale was used in the original format.

## Performing the Tests and Analysis

We have compared the acquisition of dementia knowledge by the two groups using the pre-intervention and post-intervention tests described above. The control group was fifth-year medical students in 2014 and was tested in 2014. The experimental group was fifth-year medical students in 2015 and was tested in 2015. Both groups of students took the pre-intervention test on the first day of their clinical psychiatry rotation. Then, they took the knowledge post-intervention test on the last day of their clinical psychiatry rotation.

Students who could not cooperate with the tests in this study were to be excluded, but all participated. As students who just started clinical training, we regarded the clinical ability of each student at this time as having no significant difference. Therefore, students from April to July, who had just finished 1 year and graduated to the next, were studied (in Japan, the academic year begins in April).

The statistical software used for analysis was SPSS (IBM). Independent *t* test analysis was performed to evaluate the differences between groups.

## Results

The control group had contact with actual patients and attended lectures during the rotation period. The experimental group used a simulator and had contact with actual patients as well as VPs and attended lectures during the rotation period. We carried out pre-intervention and post-intervention tests with the 43 medical students (control group) and the 36 medical students (experimental group).

On the first day of psychiatric clinical training, we administered a pre-intervention test to both groups, and mean scores were 8.42 for the control group and 8.17 for the experimental group. No significant differences were observed between the two groups’ scores, *t*(77) = 0.297, *p* = 0.767 > 0.05. On the last day of their clinical psychiatry rotation, we administered the post-intervention test, and the mean scores for the control group and the experimental group were 15.51 and 18.08, respectively. On the post-intervention test, the experimental group had significantly higher scores, *t*(77) = 2.627, *p* = 0.01 (Table 1).

**Table 1:** Means and standard deviations (SD) of the pre-intervention and post-intervention tests

	Control group (*n* = 43)	Experimental group (*n* = 36)	*P* value
	Mean (SD)	Mean (SD)	
Pre-intervention test	8.42 (4.03)	8.17 (3.39)	0.767
Post-intervention test	15.51 (4.32)	18.08 (4.35)	0.010

In addition, we administered the ARCS with the 36 medical students in the experimental group before and after they used the simulator, as a measure of motivation. Mean values on the ARCS before the students used the simulator were 24.69, 27.17, 24.83, and 27.42 for the four main categories (attention, relevance, confidence, and satisfaction), respectively. After the simulator experience, the mean values rose to 31.06, 31.00, 28.53, and 29.56, respectively. All four increases in ARCS values were statistically significant: *t*(35) = 6.163, *p* = 0.000 for attention, *t*(35) = 5.704, *p* = 0.000 for relevance, *t*(35) = 4.055, *p* = 0.000 for confidence, and *t*(35) = 2.894, *p* = 0.007 for satisfaction (Table 2).

**Table 2:** Means (M) and standard deviations (SD) of the ARCS, before and after using the software (*n* = 36)

Before using the software			After using the software			
	M	SD	M	SD	*T* value	Significance
A: Attention	24.69	4.93	31.06	3.72	6.163	0.000
R: Relevance	27.17	4.14	31.00	3.01	5.704	0.000
C: Confidence	24.83	4.04	28.53	4.57	4.055	0.000
S: Satisfaction	27.42	3.61	29.56	3.88	2.894	0.007

Furthermore, after the experimental group (36 medical students) used the simulator, the mean score on the SUS was 68.82 (standard deviation = 9.83).

## Discussion

### Impact of the Study

In this study, we compared our software with conventional learning methods as methods of acquiring the knowledge and experience required for the practice of psychiatry. On the post-intervention test, the experimental group had significantly higher scores than the control group. Moreover, after medical students used the software, their motivation scores on the ARCS significantly increased in all four main categories.

### Others’ Work

VP-based training materials clearly address the two problems mentioned above (i.e., concerns about causing harm to patients and enabling students to interact with a wide range of patient types). How well they address the third issue (the use of SPs as an alternative is limited by the cost of employing actors, their ability to adequately act out various diseases or symptoms effectively, and the need to coordinate learning times), by serving as an acceptable alternative to SPs without the logistical or performance limitations inherent in using SPs, depends on the quality of the software. In some software applications, VPs can change their facial or bodily expressions [10, 11], whereas in others, they are only static images [12]. VPs in our software can also move dynamically. In these three respects, our software has the same or better features as software developed and examined in preceding studies.

Most software developed in previous research was used for training in the interview processes. Some VPs ask questions to the learner about their illness, but software giving the experience of medical practice in its entirety is rare.

### Larger Implications

Our simulator runs in a web browser and has been developed on the assumption that students will use it over the internet. We intend to publish it on the internet in the near future, as a web-based simulator that will allow students to learn at any time and any place [21].

Our results indicate that the simulator is a promising way of solving the problems associated with the limitations of previous VP systems and improving the quality of education in the field of psychiatry. To move beyond this successful pilot study, we intend to take three steps: confirm learning effects with another cohort of fifth-year medical students, create a sufficient number of VPs with a variety of typical diseases and add ambiguous cases that would be closer to real clinical practice, and publish the software on the internet.

### Evaluation of Measures

We checked how much knowledge acquired by being in contact with actual patients improves with the use of software (VP in the examination room). However, by simulating experiences, it is expected that not only knowledge but also skills in medical care will be improved. We think that the evaluation of skills is also a necessary perspective. For this study, we measured the knowledge of the students. There was no confirmation method with the current data. In further work, we would like to confirm changes in medical skills if possible.

We attempted to ensure that the test does not favor the simulator. The test examined what students should learn through in contact with actual patients during their rotation. The four types of dementia are fundamental diseases that are questioned in national medical examinations. If students do not experience them, they have to study the material themselves during the practice period. It is possible for some students to encounter dementia often in their rotation, while other students do not experience the disease at all. It cannot be ensured that there is strictly no bias. The study has limitations on this point.

Some ARCS are measured in a scale ranging from point 1 to point 7. However, the Japanese version is given in a scale ranging from point 1 to point 9, and we used this version [17]. In the experimental group, just before the software was used, the motivation for learning was measured. Immediately after the software was used, the motivation for learning was measured. Students encountered traditional learning methods (actual patient and lecture) before using the software. At first, we thought that changes in motivation could be measured using this method. However, for motivation, it was clearer to compare the control group and the experimental group.

For cultural reasons, students unconsciously tend not to express extreme opinions in the ARCS model motivational testing. Significant results were shown in the motivational tests performed before and after the use of the software, but better results may be obtained if similar investigations are made in other countries. On the other hand, there is a background that the number of patients with dementia is increasing in Japan, so student interest is high, which may have influenced the motivation improvement. It is necessary to confirm whether the motivation for learning has been raised by creating VPs of other diseases.

### Evaluation of Virtual Patients

The results of this study suggest the potential of our simulator to improve education in the field of psychiatry. However, students worked with only four VPs (all with types of dementia) in this study. Hence, more VPs with a wider range of psychiatric disorders should be created, and the effectiveness of this simulator with this greater number and range of VPs should be examined.

In addition, the diseases in the current simulator version are all typical cases of dementia, and many atypical cases are observed in actual clinical settings. In this respect, the current version of our simulator does not reproduce real clinical practice. However, because students in clinical training do not yet have any clinical experience as doctors and have limited time for learning, we believe that it is pedagogically appropriate that the first VPs they encounter should exhibit relatively typical forms of disease. As we develop the simulator further, we plan to create a sufficient number of VPs with a variety of typical diseases and to add ambiguous cases that more closely resemble real clinical practice.

### Room for Software Improvement

We acknowledge that the simulator still has room for improvement. Some students indicated areas of potential improvement in their comments on the feedback form, such as the following: “The prescriptions were difficult,” “I wanted to know how to increase my score,” “I wanted to experience cases other than dementia patients,” and “I wanted to ask open-ended questions.” The technology does exist that would permit students to ask their own questions freely using a voice recognition system, but currently our simulator offers only a choice from a predetermined set of questions as inputs. Because of the limitations of current voice recognition technology, the probability that the simulator would provide appropriate responses to questions inputted in that way is approximately 70% [10]. If the responses are inadequate because of recognition errors, students will become less willing to use the software [13]. For this reason, we believe that the introduction of free text input (i.e., questions independently generated by the student) should be deferred to a future time.

Although this measurement was unrelated to the study’s main objective, the average SUS score of students who used our simulator was 68.82. This score is in the high marginal range, indicating that the simulator is approaching the performance level of an acceptable system. The SUS dictates that only a score above 70 should be considered acceptable. However, a cultural factor may have affected the results. We used a direct translation of the scale into Japanese, which did not take into account differences caused by subtle cultural nuances. Japanese people tend not to express extreme opinions, relative to other cultures; therefore, they are likely to have avoided selecting “strongly agree” or “strongly disagree,” even if they had strong feelings regarding an item. This tendency would push the average score of SUS closer to the central value of 50.

### Future Development

The acquisition of psychiatric knowledge (on dementia) and the increase in motivation associated with use of the comprehensive clinic simulator were satisfying results. However, we intend to confirm the positive learning effects with the upcoming year’s cohort of fifth-year medical students, as overall student achievement can sometimes vary between cohorts depending on the atmosphere of the academic year.

This study shows the possibility that our comprehensive clinic simulator can improve psychiatric education. Accordingly, we look forward to pursuing further confirmation of its learning effects, developing additional VP cases, and making the software available to medical students as widely as possible.

## Abbreviations

CBT: Computer-based testing
OSCE: Objective Structured Clinical Examination
IRT: Item Response Theory
SPs: Standardized patients
VPs: Virtual patients
SUS: System Usability Scale
M: Means
SD: Deviations
ARCS: Attention, Relevance, Confidence, and Satisfaction
